# Vitamin C for attenuating postherpetic neuralgia pain: an emerging treatment alternative

**DOI:** 10.1007/s10194-012-0476-z

**Published:** 2012-08-30

**Authors:** Shailendra Kapoor

**Affiliations:** 74 Crossing Place, Mechanicsville, VA 23239 USA

To the editor,

I read with great interest the recent article by Sayanlar et al. [1] in a recent issue of your esteemed journal. Interestingly, the past few years has seen the identification of a number of alternative treatments for postherpetic neuralgia.

One such emerging alternative is vitamin C. Vitamin C attenuates spontaneous pain in patients with postherpetic neuralgia [2]. This has been confirmed in recent studies. For instance, Schencking et al. [3] in a recent multicenter cohort study have demonstrated the effectiveness of vitamin C in herpes zoster treatment. Vitamin C acts by modulating serum levels of cytokine IL-6 and IL-8.

Vitamin C is especially useful in patient with recalcitrant postherpetic neuralgia resistant to standard therapy. Resolution of dermatologic Zoster lesions is seen in as less as 10 days following intravenous administration of ascorbic acid [4]. Interestingly, the patients afflicted with postherpetic neuralgia demonstrate lower levels of ascorbic acid [5].

Clearly, vitamin C has significant potential in mitigating postherpetic neuralgia, especially when administered intravenously. Hopefully, the coming few years will see the increased use of vitamin C in the treatment of herpes zoster, especially in treatment resistant patients.
